# Cytogenetics and Consequences of Polyploidization on Different Biotic-Abiotic Stress Tolerance and the Potential Mechanisms Involved

**DOI:** 10.3390/plants11202684

**Published:** 2022-10-12

**Authors:** Md Mazharul Islam, Deen Mohammad Deepo, Saifullah Omar Nasif, Abu Bakar Siddique, Oliul Hassan, Abu Bakar Siddique, Narayan Chandra Paul

**Affiliations:** 1 Department of Horticultural Science, Kyungpook National University, Daegu 41566, Korea; 2 Research and Development, Horticultural Crop Breeding, Quality Feeds Limited, Dhaka 1230, Bangladesh; 3 Global Centre for Environmental Remediation (GCER), College of Engineering Science and Environment, The University of Newcastle, Newcastle, NSW 2308, Australia; 4 Cooperative Research Centre for Contamination Assessment and Remediation of the Environment (CRC CARE), ATC Building, The University of Newcastle, Newcastle, NSW 2308, Australia; 5 Department of Plant Physiology, Umeå Plant Science Centre, Umeå University, 90736 Umeå, Sweden; 6 Department of Ecology and Environmental System, College of Ecology and Environmental Sciences, Kyungpook National University, Sangju 37224, Korea; 7 Department of Plant Biology, Swedish University of Agricultural Sciences, 75007 Uppsala, Sweden; 8 Kumho Life Science Laboratory, Department of Integrative Food Bioscience and Biotechnology, Chonnam National University, Gwangju 61186, Korea

**Keywords:** cytogenetics, stress, polyploidy, fluorescent in situ hybridization, genomic in situ hybridization

## Abstract

The application of polyploidy in sustainable agriculture has already brought much appreciation among researchers. Polyploidy may occur naturally or can be induced in the laboratory using chemical or gaseous agents and results in complete chromosome nondisjunction. This comprehensive review described the potential of polyploidization on plants, especially its role in crop improvement for enhanced production and host-plant resistance development against pests and diseases. An in-depth investigation on techniques used in the induction of polyploidy, cytogenetic evaluation methods of different ploidy levels, application, and current research trends is also presented. Ongoing research has mainly aimed to bring the recurrence in polyploidy, which is usually detected by flow cytometry, chromosome counting, and cytogenetic techniques such as fluorescent in situ hybridization (FISH) and genomic in situ hybridization (GISH). Polyploidy can bring about positive consequences in the growth and yield attributes of crops, making them more tolerant to abiotic and biotic stresses. However, the unexpected change in chromosome set and lack of knowledge on the mechanism of stress alleviation is hindering the application of polyploidy on a large scale. Moreover, a lack of cost–benefit analysis and knowledge gaps on the socio-economic implication are predominant. Further research on polyploidy coupling with modern genomic technologies will help to bring real-world market prospects in the era of changing climate. This review on polyploidy provides a solid foundation to do next-generation research on crop improvement.

## 1. Introduction

The duplication of single or combined differentiated genomes is known as polyploidy. Autopolyploid arises from the doubling of structurally similar, homologous (AAAA) genomes within a single species, while allopolyploids arise through interspecific hybridization and subsequent doubling of nonhomologous (AABB) genomes [1,2,3]. Aneuploid genomes have incomplete chromosome sets, which occur naturally in plant populations and are induced by chemical and physical agents [4,5,6]. Differences in the number of chromosomes [7,8,9] and phenotypic differences [10,11,12] have been verified using molecular cytogenetic methods. These differences increase allergic diversity [13] and heterozygosity [14] by genome buffering, resulting in novel dose effects. For instance, wheat, canola, cotton, peanut, soybean, and tobacco [1,6,15,16] have been identified as domesticated crops.

Several chemical and gaseous agents are currently used to induce polyploidy. The most widely used agents are colchicine and oryzalin, though colchicine has been suggested to be avoided due to its carcinogenic properties [17]. The common consequences of induced polyploids are increased cell size as well as whole plant size, reduced fertility, and heterosis [18]. In modern breeding, cytoplasmic male sterility often occurs in F1 progenies, where N_2_O gas treatment (~6 bar, 48 h) plays a significant role in restoring fertility [19]. Similarly, pre- and post-fertilization barriers can be minimized through embryo rescue, particularly in flowering ornamentals, such as *Lilium and Hibiscus* [20,21,22,23]. Currently, polyploidization is vital in creating crop diversity and producing fruits, vegetables, and flowers most sought after by consumers.

Due to climate change and global warming, plants experience multiple abiotic stresses such as salinity [24,25,26,27], drought [28,29,30], temperature [31,32,33,34], and biotic stresses such as insect pests [35,36,37] and diseases. These stresses threaten crop production by disrupting plants’ physiological and biochemical processes. Polyploidy offers several advantages amid stress situations, which are described in previous studies. However, the underlying mechanism and holistic understanding are still missing. 

Several studies assessed polyploidy, but cytogenetic evaluations of polyploids and their effects on crop physiology remain uncertain. Therefore, polyploidy’s roles in plant development, mechanisms and assessment processes, plant physiology changes, biotic-abiotic stresses, challenges, and prospects are outlined here. This study aims to help to recognize possible interactions between polyploids and to establish a consistent assessment. 

## 2. Role of Polyploidy in Modern Plant Breeding

Plant breeders modify crop traits using multiple tools, including polyploidization, to satisfy market demand. This technique creates intense phenotypes and high vigor, making it one of the most potent crop enhancement methods [38]. In addition, some plants have a specific demand for their specific traits, such as seedless fruits in grapevine and banana, which can be achieved through polyploidization [39,40]. Polyploidy results in higher heterozygosity and genome redundancy that are considered advantageous for improving crop plants over conventional plant breeding tools [41,42].

Interspecific hybridization helps to increase the diversity of crops and helps them adapt to new environments [43]. For example, Allopolyploid Triticale is a manufactured crop developed by crossing hexaploid bread wheat and rye to achieve specific goals (e.g., high yield, grain quality, less disease, and stress tolerance) [44,45]. In addition, bridge hybridization is done to transfer genes from one ploidy stage to another if a direct crossover is not feasible. Creation of diversity is one of the most important tasks to develop a crop variety where polyploidy has the efficacy to enhance crop diversity [46,47]. 

Polyploidy is common in newly domesticated crops [48]. In many cultivated crops, polyploidy has been observed in the speciation process and is now commonly used to create new species selected for features [3]. Unreduced gametes result in plant polyploidy and are used in crop breeding. Polyploidy increases the chromosome number, which helps plants tolerate the mutation by allelic modifications [49,50]. Chromosome deletion-related polyploidy breeding and substitution can produce targeted traits. We have seen many examples of successful cultivation influenced by polyploidy breeding. For example, seedless triploid watermelons, tetraploid red clovers, ryegrass, rye, and many ornamental plants have been developed or improved using polyploid breeding [17,51].

In summary, the main benefits of polyploidy are related to improving the use of heterozygosity. It buffers the effect of gene redundancy in mutations and, in some cases, facilitates reproduction by self-fertilization or asexual means [52]. It has a significant influence on farmers and food security issues.

## 3. Induction of Polyploids

The recurrence and frequency of polyploidization in plant species make polyploidization an influential research area [53] in which a major step is to select traits in plants [54]. The occurrence of polyploidy in plants was discovered about a century ago. Because of the widespread occurrence of polyploids in wild and cultivated plants, it is important for plant breeders and evolutionary biologists. In the past, antimitotic reagents-induced polyploids have not directly contributed to crop improvement. On the other hand, sexual polyploids (unreduced 2n gametes) are more relevant for crop improvement in many cases. Two pathways cause polyploids: mitotic polyploidization and meiotic polyploidization [55].

**Figure 1:**
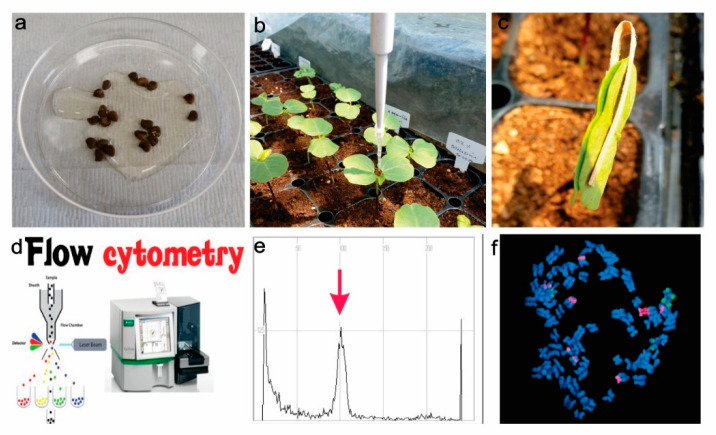
Mechanism of in vivo polyploidization; (**a**). Seeds soaking (6–24) hours with colchicine (0.01–0.2)%, (**b**). Colchicine treatment (10–20 µL) 10 days in the young leaves, (**c**). Leaves binding with clips for maximum chemical attachment, (**d**). Flow cytometry analysis for ploidy level assessment, (**e**). Ploidy level assessment by a histogram, (**f**). Hibiscus ploidy assessment using chromosome number; and 5S rDNA (green) and 18 rDNA (red) signals.

Mitotic polyploidization depends on doubling somatic tissue where homoeologous chromosome recombination occurs [56]. The first mitotic polyploidization was introduced in 1930 [57]. This activation polyploidization was tested on plants in vitro [55]. Colchicine, oryzalin, trifluralin, amiprophos-methyl, N_2_O gas treatment, and caffeine have recently been used as antimitotic reagents [17]. Colchicine is an alkaloid from wild meadow saffron and was the most used as an antimitotic reagent. Oryzalin is a potent herbicide from the Dow AgroScience, USA toluidine chemical band [17,58]. Wetting roots or auxiliary buds or shoots with a colchicine solution of a specific concentration and duration resulted in the successful development of polyploids in many crop species [57,59], as shown in Figure 1. Previous studies successfully applied in vitro chromosomes doubling of colchicine and oryzalin for starch, fodder beet, ryegrass, oriental melon, watermelon, and red clover [17,60].

In vitro polyploidization showed better performance than the success rate of in vivo polyploidization in sugar and fodder beet, ryegrass, and red clover [55,59]. Table 1 provides a list of crops, vegetables, and ornamental and medicinal plants treated with chromosome antimitotic agents for chromosome duplication using different methods and protocols.

Meiotic polyploidization produces 2n gametes due to the incomplete division of chromosomes [86]. Polyploids that originate through the functioning of 2n gametes are called sexual polyploids, and their usefulness for crop improvement has been demonstrated in potato, alfalfa, and red clover. Introgression can be accomplished by recombination due to genetic crossing-over between alien chromosomes as well as the addition of alien chromosomes in the case of sexual polyploidization in allopolyploids, which is exceedingly difficult or unlikely in the case of colchicine or oryzalin induced allopolyploids. This deviation can occur in plants with normal chromosome pairing as well as in those with disturbed chromosome pairing such as homoeologous recombination of meiotic replication that was seen in *Alstroemeria* [87], *Lilium* [88] and *Gasteria lutzii* × *Aloe aristate* [89]. The process leading to the formation of 2n gamete is called meiotic nuclear restitution during micro- or megasporogenesis. Depending on the meiotic stage at which nuclear restitution occurs, different restitution mechanisms have been recognized, such as first division restitution (FDR), second division restitution (SDR) [90], and novel intermediate meiosis restitution [88]. In FDR, the non-sister chromatids are heterozygous from the centromere to the first convergence point, while preserving heterozygosity in both parents [91]. In SDR, the two sister chromatids are homozygous between the centromere and the first crossover point, and the resulting gametes have lowered heterozygosity levels compared to the parents [92]. In some cases, 2n gametes restitution cannot be classified as FDR or SDR; the word “indeterminate meiotic restitution” (IMR) has been coined to describe it [88]. Furthermore, IMR might be a widespread occurrence in allotriploids, where both bivalents and univalents are most produced.

## 4. Cytogenetic Evaluation of Induced Polyploids

Traditionally, polyploids have been assessed by morphological examination. Advanced cytogenetic methods such as flow cytometry, genomic in situ hybridization (GISH), and fluorescence in situ hybridization (FISH) are currently used for polyploid evaluation [11,20,22,93].

### 4.1. Flow Cytometry

Counting chromosomes in an individual cell is the most efficient and accurate way to confirm ploidy. However, the basic number of chromosomes must be identified before counting. Furthermore, chromosomes are confusing regarding mixoploidy because of the smaller size with a higher number of chromosomes, e.g., taxa *Hibiscus* [94] and taxa *Chrysanthemum*. 

**Figure 2:**
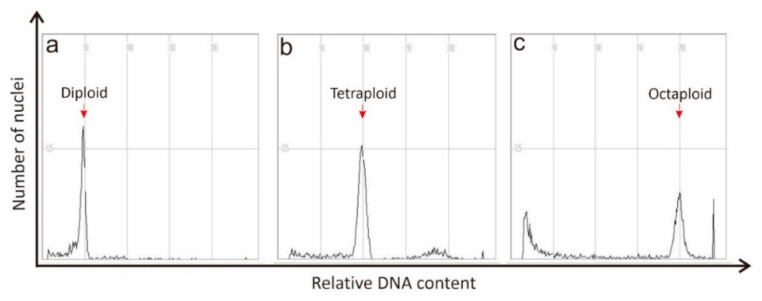
Histograms show the flow cytometry analysis of comparative changes in ploidy levels in watermelon. (**a**) Diploid (2*n* = 2*x* = 24), (**b**) tetraploid (2*n* = 4*x* = 48), and (**c**) octaploid (2*n* = 8*x* = 96). Red arrows indicate ploidy levels of diploid, tetraploid and octaploid.

Flow cytometry may also be the unique method incorporating strong analytical utility to calculate the cell nucleus’s physical size and genome [95,96]. Flow cytometry is a comparatively simple and easy method of calculating a polyploid’s nuclear DNA material. By measuring the relative DNA content using flow cytometry [97,98], the ploidy level of mediated polyploids can be easily verified (Figure 2). However, flow cytometry has some limitations as it is not precise enough to estimate the exact chromosomes number and is unable to differentiate the variation in chromosomes number compared to the ploidy level.

### 4.2. In Situ Hybridization

**Figure 3:**
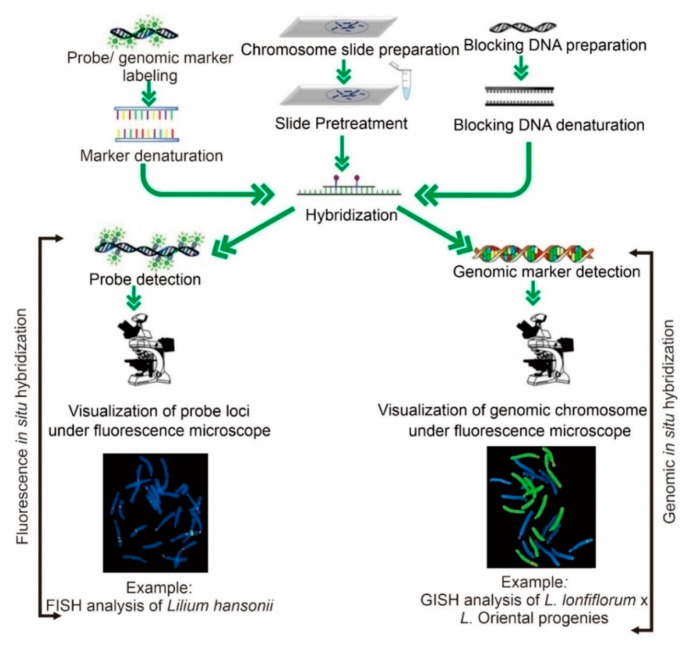
Working steps for fluorescent and genomic in situ hybridization were used for the cytogenetic study of horticultural modified crops. Different methods, such as nick translation, random primed labeling, and PCR, are used to label the probe during marker labeling. Various methods, such as autoclaving, shearing the DNA with a tiny needle in a syringe, or sonicating, are used to prepare to block DNA. Chromosome slide preparation is the selection of well-spread chromosomes prepared from a young root tip using an enzyme mixture at 37 °C. Slide pretreatment is the enzymatic digestion of the chromosomes in order to unmask the DNA prior to hybridization. Hybridization involves the attachment of blocking and probe/genomic markers with chromosomes to identify the specific loci/origin of the genome of the respective chromosome. During detection, attachment of the designed antibody against the target marker along with blocking buffer to detect the specific fluorochrome.

Molecular cytogenetic research approaches such as FISH and GISH are commonly used and well-respected tools to investigate plant genetics. FISH and GISH have often been used to identify information surrounding chromosomal mutations, structure, and genomic evolution [99,100]. Oligos specific to a repetitive sequence or a particular genomic region can be visualized using fluorescence in situ hybridization [8,101,102,103]. For example, different ribosomal DNA (rDNA) signals are doubled in a tetraploid compared to those in a diploid. A study of 5S rDNA in cotton plants revealed that most diploids have two 5S rDNA signals and all allotetraploid species have four 5S rDNA signals [104]. The same result was found in a woody species of the genus *Rubus* [105]. The 45S, 18S, 25S, and 5S rDNA are commonly used as FISH markers for cytogenetic study. This method has been used intensely in gene duplication methods and amplification in intraspecific and interspecific polyploids. A brief working directory of FISH and GISH is shown in Figure 3.

**Figure 4:**
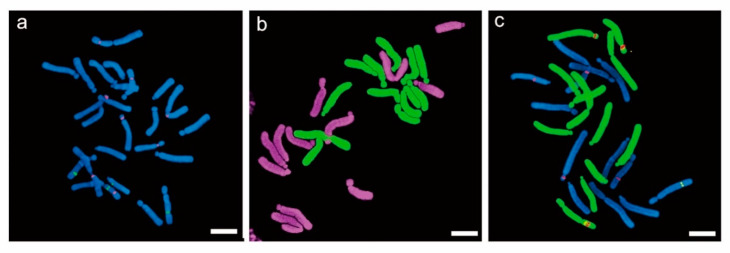
In situ hybridization of diploid (2*n* = 2*x* = 24) Lilium. (**a**). FISH analysis of intraspecific F1 using 5S and 45S ribosomal DNA; (**b**). GISH analysis of interspecific (*L. longiflorum* × *L. hansonii*) F1 using genomic DNA; and (**c**). FISH and GISH combined analysis of interspecific (*L. longiflorum* × *L*. Oriental hybrid) F_1_ using 5S, 45S rDNA, and genomic DNA.

GISH is also useful for studying cytogenetics and determining hybridity status, particularly in the case of interspecific plant hybridization [22,106,107,108,109]. Within an interspecific polyploid, GISH distinguishes the genomic structure, chromosomal constituents, crossing over, aneuploidy, and alien genes introgression. For example, in diploid interspecific Lilium (*L. longiflorum* × Asiatic lily; 2*n* = 2*x* = 24), 12 *L. longiflorum* and 12 Asiatic chromosomes can be identified using GISH (Figure 4). The number of *L. longiflorum* and Asiatic chromosomes are doubled in the induced tetraploids and can be visualized through GISH. Therefore, GISH is an advanced multicolor detection technique that plays a gratuitous role in the chromosomal and genomic investigation of induced polyploids.

## 5. Effect of Polyploidization at the Morphological and Molecular Level

Polyploidization results in morphological changes in plants due to whole genome duplication, changes in chromosomal structure, nuclear enlargement along with gene dosage and epigenetic consequences, as well as an increased number of larger cells [18,110,111,112,113,114]. Further, due to the changes in different levels, several morphological traits such as plant height, root length and number, leaf number, area and size, pollen size and number, stomata number and size, and flowers and fruits number and size as summarized in Table 2.

**Figure 5:**
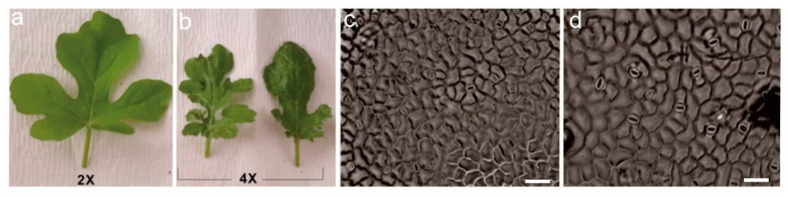
Leaf morphology and stomata size of watermelon induced by oryzalin. (**a**). diploid leaf, (**b**). tetraploid leaf, (**c**). stomata of diploid, and (**d**). stomata of a tetraploid leaf, respectively. Scale bar= 10 μm [17].

With the increase of ploidy level, the plant height, width and length of flower, flower size, and the number of internodes in dendrobium increased [17,126,127,128]. Polyploidization affects the floral traits such as flowering time, flower diameter, shape, size, and color, as well as different parts of flowers in kiwifruit and salvia [9,129,130,131,132,133,134,135,136,137]. These shreds of evidence suggest that polyploidy can be applied in plant breeding by targeting the flower size, shape, color, modifications in size, and the number of floral parts. Fruit size and fruits number, along with other fruit characteristics such as fruit weight, fruit peel, flesh weight and seed number are affected by the increase of ploidy number [130,138,139,140,141,142,143,144]. Due to variations in cell size and chromosome size (Figure 5), polyploidy changes the characters of the leaf [145,146,147,148,149,150,151,152,153]. Stomata number, density, size, and area are the important traits of leaves that are affected by the change of ploidy number [154,155], and this effect (Figure 5) has been observed in citrus [120].

Changes in fruits, leaves, flowers, and color can be considered from the application point of view. Targeted traits can be achieved along with higher variation with the changes of ploidy level. A change in ploidy level also affects molecular and gene expression. Changing the ploidy level due to changes in nuclear DNA, chromosome number, and structure can manipulate genetic diversity, genome replication, gene expression, and heterosis [156]. Changes in ploidy level affect DNA content and the number of chromosomes [157,158,159]. During replication, polyploidization often induces epigenetic changes such as transposon simulation and chromatin modification, as well as the extension or loss of chromosomal fragments. The polyploidization effect at the plant morphology, physiology, and molecular levels needs extensive research to reveal the mechanisms that will help plant breeders for directed modification and crop improvement.

## 6. Effect of Polyploidization on Abiotic Stresses

### 6.1. Salinity Induced Stress Alleviation

H_2_O_2_ and malondialdehyde (MDA) concentrations increase in salinized tissues due to the generation of reactive oxygen species (ROS) [160,161]. Proline plays a pivotal role in alleviating salt-induced stress by maintaining cell turgor (i.e., as osmolyte) [162]. Polyploids reduce the H_2_O_2_ and MDA concentrations, increase proline concentration, and tolerate salinity stress. Higher proline concentration and lower H_2_O_2_ and MDA concentration (Table 3) in polyploid plants are reported in the studies [163,164]. Due to MDA’s lower concentration in tetraploids-maintained cell membrane integrity, and Na+ hardly reached the cells. Conversely, higher H^+^ transport through cells in tetraploid rice cultivars may be attributed to salt tolerance. Interestingly, Tu and coworkers [163] noticed that a defensive space between the pericycle and cortex contributes to more salt tolerance. Further, Jinag et al. [164] reported that the mortality rate of tetraploids in saline stress was 12.3–12.6% lower than that of diploid ones (Table 3). Meng et al. [165] reported that tetraploids show a stable K^+^/Na^+^ ratio (16:10 and 15:10, respectively, in roots and shoots), while K^+^ decreased in diploid turnips (46:100, and 48:100, respectively, in roots and shoots). Diploid turnips also experienced a significant reduction in chlorophyll content (40.3% versus 11.9% in tetraploids). Furthermore, seed germination, root, and shoot growth were enhanced in polyploid during salt-induced stress (Table 3) [163,165,166,167]. Although salinity has a more extreme effect on diploids than on their corresponding tetraploids, the underlying mechanism in tetraploid plants is unclear. Besides, no study was conducted on actual saline containing different salt solution mixtures in different concentrations. Thus, tetraploid behavior in natural conditions is difficult to predict. Table 3 shows the salinity-inducing methods, test crop, polyploidy adaptation, and effect on test crop.

### 6.2. Drought Stress Alleviation

Previous ploidy-level research has suggested that enhancing ploidy can successfully alleviate or help plants better adapt to drought stress, as depicted in Table 3. Due to osmotic stress effects, cell plants usually incur damage by producing MDA and other superoxide’s that cause cell membrane disintegration. Tetraploid plants showed lower MDA concentrations for drought tolerance than diploid plants [168,171,173]. Although diploids and tetraploids experience increased ROS production due to drought stress, ROS scavenging and ROS homeostasis increased in tetraploids [171,172]. Moreover, Yang et al. [131] found more superoxide dismutase (SOD), peroxidase (POD), and catalase (CAT) in tetraploid ones during drought stress. Similarly, 30–40% more phenolic content and higher antiradical activity are found in tetraploid cultivars than in diploid cultivars, indicating ROS homeostasis and better tetraploid stress adaptation [172].

To mitigate drought stress, plants undergo stomatal leaf closure to reduce transpiration. Enhanced ABA in plants, especially in leaves, reduces leaf turgor, resulting in decreased stomatal pore aperture, which reduces the incidence of leaf water loss. Eventually, plants can conserve water within themselves. Polyploid plants show more ABA synthesis than diploids when under drought. Detailed work has been carried out by Rao et al. [169], establishing that polyploid plants show more drought stress synthesis than diploids. In addition, some ABA expresser enzymes such as 9-cis-epoxycarotenoid dioxygenase 1 (NCED1), NCED2, and gene expression ABRE binding factor 5-like (ABF5-like) were observed; such phenomena in tetraploids are responsible for increasing ABA synthesis and signaling pathways for stress adaptation. On the other hand, aquaporin genes such as MdPIP1;1 and MdTIP1;1, which are responsible for cell-to-cell water transportation, are expressed less in polyploid plants ([168] Table 3). Overall, morphological growth and chlorophyll content in tetraploids were higher than in diploids during drought stress [168,169,170,171,172] (Table 3). Nevertheless, consideration should be given to the relative fitness of different ploidy levels at different drought levels. In addition, to evaluate the efficacy of polyploidy in drought tolerance, other environmental factors associated with drought stress are also important.

### 6.3. Temperature Stress Alleviation

Extreme temperature escape by polyploidy has certain trade-offs. Chen et al. [177] experimented on the effects of heat stress on diploid and tetraploid *Asparagus officinalis* by putting both cultivars under extended stress. They placed both cultivars at 45 °C for 96 h and observed better adaptation of tetraploid plants than diploids (Table 3). The tetraploid cultivar also had higher photosynthetic pigments and lower stomatal densities than the diploid. Zhang et al. [53] worked on diploid and tetraploid *Dioscorea zingiberensis* and found similar results from tetraploids under temperature stress. Usually, in temperature stress, due to ROS production, plants experience decreases in the level of ascorbate (AsA) and glutathione (GSH). Polyploid *Dioscorea zingiberensis* showed a gradual instead of a drastic reduction in diploids in these antioxidant compounds [53]. This result is strong evidence of tetraploid heat-stress alleviation. However, Godfree et al. [174] proposed that polyploidy is not solely responsible for stress adaptation. They suggested that both polyploidy and reproductive homeostasis contribute to heat stress alleviation. They found distinct morphological differences, consistently heavier seeds, and decreased seed sizes in tetraploids than diploid, which they considered stressful reproductive production homeostatic maintenance. To understand how polyploidy affects transcriptomic responses to temperature stress, Yin et al. [178] experimented with a diploid and a tetraploid *Dioscorea zingiberensis*. They found “Activation Transcriptomic Reaction” in tetraploids, in which 19 bands were silenced and 47 bands were activated in diploids, 32 bands were silenced, and 28 bands were activated under temperature stress. They reported that enhanced transcriptomic responses to activation could confer tolerance in tetraploids during heat stress. Alternatively, Liu et al. [175] observed varied heat and cold stress responses from tetraploids. They calculated the LT_50_ (lethal time to 50% plant mortality under stress) and found that tetraploid LT_50_ was 2.40 times lower than that of diploids in cold conditions. Although in heat stress, diploid LT_50_ was 1.20 times higher than tetraploid, which suggests lower tetraploid heat stress tolerance. Such varied results and findings from various research indicate the need for further research on temperature stress. Information regarding polyploidy-induced abiotic stress alleviation is presented in Table 3.

## 7. Effect of Polyploidization on Plant Biotic Stresses

### 7.1. Polyploid-Insect Interaction

While plant polyploidy on insect abundance and dispersal is uncertain, two major events are observed in insect physiology, such as i. polyploidy caused novel insect defense (herbivores), and ii. co-opt counter-insect defense and extended host selection [178,179,180]. One example of a polyploidy-induced defense mechanism can be seen in Brassicales, in which genome duplication contributes to the development of glucosinolate compounds to establish protection against butterflies [180]. However, Edger et al. [180] also stated that, in some cases, herbivores are drawn to polyploids through coevolutionary mechanisms. Polyploidy shows different effects on herbivory, often divided into attraction and escape. Concerning attraction, Arvanitis et al.’s [178] findings provide adequate evidence. In a common garden where the corresponding tetraploid and octoploid Cardamine pratensis were grown, bud gall midge *Dasineura cardamine* preferred octoploid over tetraploid where tetraploid cardamines rarely struck. Does higher polyploidy attract insects? Herbivores are typically fond of polyploids. Their argument is backed by Thompson et al. [181], who recorded higher infestations of prodoxy moth *Greya politella* in *Heuchera grossulariifolia* tetraploids compared to its diploid ones. Similarly, in tetraploid *Arnica cordifolia* [182], tephritid fly *Campiglossa footeorum* displayed higher attack rates than triploid cultivars.

**Figure 6:**
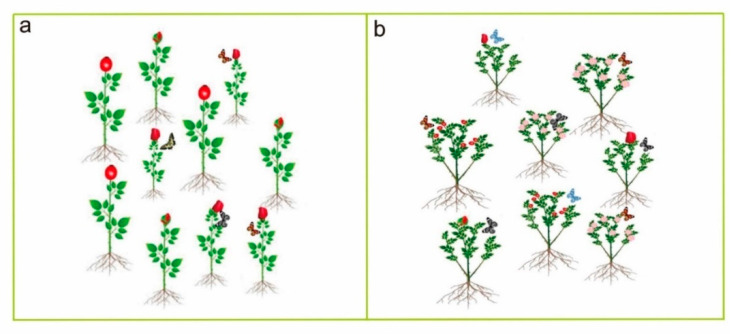
Can insects identify different ploidy level plants? According to Segraves and Anneberg [185], insects forage more on predominant cytotypes in a natural habitat where different ploidy plants coexist ((**a**); where we imagine that small plants are predominant here, insect will forage more on small plants rather than flowers of bigger plants, i.e., irrespective of ploidy level). Contrarily, insects forage equally in a common garden where mixed cytotypes are grown ((**b**); imagine there are different ploidy levels flower in the common garden. Insects generally fail to detect different ploidy levels; thus, they forage equally in a common garden).

On the other hand, Nuismer and Thompson [183] recorded frequent attacks by a stem borer moth *Greya piperella* in *Heuchera grossulariifolia* diploids rather than in tetraploids. Likewise, diploid *Gymnadenia conopsea* orchids [184] were more frequently attacked by aphids than their corresponding tetraploids. These contrasting findings indicate that polyploidy does not inhibit the behavior of herbivores. However, it has been suggested that insect herbivory is not a cytotype-dependent habitat selection but plays a key role in host-seeking. In open fields and natural ecosystems, insects typically prefer the most common host in that habitat. Alternatively, in a typical garden (Figure 6) where all cytotypes are grown, insects forage the hosts equally [185]. Although the results show that polyploidy has various implications concerning insect attacks, it can provide some trade-offs. Generally, polyploids produce higher growth and reproductive ability. Therefore, polyploids can help avoid economic injury and an herbivory-induced economic threshold, which can be difficult in diploids.

### 7.2. Polyploidy and Pathogen Resistance

Pathogens, including fungi, bacteria, and viruses, are the most daunting factor for crop cultivation worldwide. The grower must account for high economic losses incurred by yield loss and the application of pesticides to combat diseases [186]. Developing pathogen-resistant crop varieties through selective breeding is crucial to address this problem. The induction of polyploidy may be a promising solution [149]. Naturally occurring varieties of different crop species, including banana, strawberry, and watermelon, have been reported to resist a broad range of pathogens [186,187]. Allotriploid cultivars of banana (AAB) and polyploid watermelon germplasm are fusarium-resistant [187]. Many cultivars of octoploid *Fragaria* × *ananassa* Duchesne are resistant to anthracnose, fusarium wilt, crown rot, red core, verticillium wilt, and angular leaf spot [186]. Polyploid strawberries (US4808 and US4809) reported resistance to four *Xanthomonas fragariae* groups [186].

Several studies have reported that pathogen resistance in the polyploid genotype is higher than its diploid generation [187,188]). Autotetraploid and autotriploid watermelon demonstrated higher fusarium resistance than diploid watermelon [187]. Diploid apple cultivars are more susceptible to *Alternaria alternata*, and *Colletotrichum gloeosporioides* than autotetraploid apple cultivars Hanfu and Gala [188]. Allopolyploid tobacco prevents plant viruses better than diploid tobacco [189]. Tetraploid wheat avoids more powdery mildew and leaf rust than diploid wheat [190,191]. Allelic diversity, gene expression (over), and physiological state are the key factors determining a host plant’s ability to withstand various pathogens.

**Figure 7:**
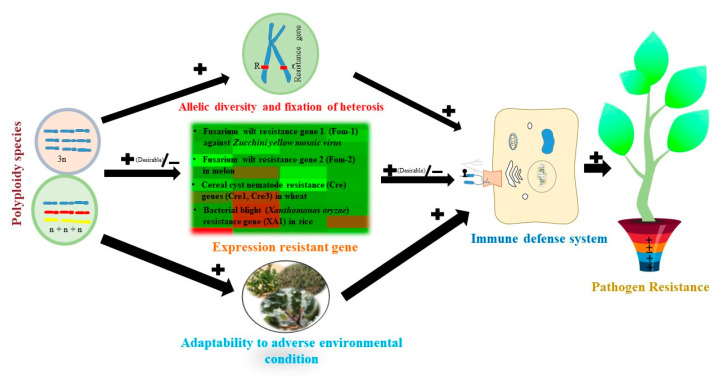
Effect of polyploidy on pathogen resistance. The sign next to the arrow gives the direction of the effect. The sign “+’’ means the higher ploidy level increases the probability of effects, while “−” means the higher ploidy level decreases the probability of effects. Pot represents the combined effect of the polyploidy plant. The gene-for-gene model is the general mechanism of pathogen resistance. In the polyploidy host, high allelic diversity with dominant allele, fixed heterosis, and high expression (desirable) of resistance gene directly influence the pathogen resistance. The validated disease resistance genes and their target pathogens are given as examples [54]. The effects of ploidy-level variation on host adaptability under diverse environmental conditions (biotic and abiotic stress) could indirectly influence parasite resistance.

Polyploidy may influence plant species’ allelic diversity, gene expression (over), and physiological condition (Figure 7). The extra alleles at a given locus in polyploids increase allelic diversity due to the high probability of heterozygosity and enhance resistance. Multiple polyploid chromosome sets increase gene expression [192,193]. In contrast, it has been proposed that gene expression is downregulated (in certain loci or in the whole genome and sometimes even silenced) with increased ploidy level [10]. Polyploid plants can adapt to a wide range of environmental conditions by developing stress tolerance [194]. Invasive plant species are more tolerant of diseases than individuals suffering from environmental stressors [195]. Therefore, the polyploid with higher allelic diversity at resistance genes, higher expression levels of immune genes should select for cultivar development. The positive relationship between the disease resistance of the plant and environmental stressors also needs to be considered.

## 8. Challenges of Polyploidization

### 8.1. Changes in Cellular Architecture

Due to an increase in an organism’s genomic content, cell volume usually increases. It has the consequent change in the relationship between the cell’s tridimensional and bidimensional components [196,197]. Doubling the genome is expected to double the amount of chromatin, but only causes a 1.6-fold increase in the nuclear envelope surface. Cell size expansion can contribute to anatomical imbalances and deleterious effects, such as gene redundancy shields polyploids from the mutations’ prejudicial effect, infertility, brittle wood, and watery fruits [198]. Moreover, polyploidization can cause albinism [199].

### 8.2. Mitotic and Meiotic Abnormalities

Normal mitosis and meiosis are frequently disrupted in polyploids. Due to additional sets of chromosomes present in an induced polyploid, faces various challenges during mitotic chromosome segregation. As we observed that the homozygosity or heterozygosity level significantly differs in polyploids. It depends on the formation pathway like autopolyploid or allopolyploid that affect the performance of the polyploids in fertility, growth and even yield [200,201]. Autotetraploid yeast demonstrates increased mitotic loss of chromosomes, producing aneuploidy cells [202]. Spindle abnormalities usually cause difficulties in mitosis. Chaotic mitotic chromosomal segregation also occurs in wild yeast [203]. However, there is little knowledge regarding the mitotic stability of polyploid plant cells. Meiosis requires three or more chromosome sets in which the frequency and manner of development of aneuploidy depend on the type of polyploidy. Triploidy and aneuploidy, which may arise from meiotically unpaired DNA, are more unstable states than tetraploidy. These frequently lead to or result from the more stable polyploidy states. Both conditions may have potentially detrimental effects on genome regulation [52]. Several experiments resulted in 30–40% aneuploidy of autotetraploid maize [204,205]. Another problem occurs in triploids and pentaploids; trivalent cannot be solved into balanced products in triploids, and a spontaneous division of multiple forms of chromosomes produces mainly aneuploid gametes. In addition, normal chromosomal segregation is another challenge in auto and allopolyploid where the multivalent complex structure is often associated. Our study found that the multivalent has an important role in abnormal chromosomal segregation reducing fertility [200,206].

### 8.3. Epigenetic Instability

Aneuploidy can cause epigenetic and genomic instability [207,208]. In autopolyploids, instability can directly or indirectly contribute to genome duplication. The proof was demonstrated when diploid and tetraploid Arabidopsis thaliana compared epigenetic effects at a transgenic locus [158,209,210]. Epigenetic changes in the gene have also been found in allopolyploids. Theoretically, *A. thaliana* has also demonstrated regulatory improvements in autopolyploid strains in parents. Such changes involved silencing or activating genes, including activating a Spa-CACTA family DNA transposon [201,210]. Therefore, mismatches in gene expression and gene product regulatory controls can decrease fitness.

## 9. Conclusions and Future Perspective

In plant breeding, polyploidization is a successful technique for developing novel traits. Polyploidy causes significant transcriptomic and regulatory changes that bring physiological and morphological changes. Disrupted regulatory factor stoichiometries, small RNAs, and other genome interactions could potentially set these in motion, cascading through entire networks of transformed regulatory modules from single-gene expression modification. Plants with duplicate whole chromosome sets have more distinctive features, such as a different phytochemical profile, higher content of desired pharmaceutical molecules, plant shape, flower color, size and style, fragrance, vase life, and extended flowering time. However, they do not always act in the same way. Furthermore, polyploid clones of *Eucalyptus grandis*, *E. urophylla* recently produced a fiber with higher length and thickness, resulting in improved paper formation and strength, suggesting that polyploids could be used in pulp and paper production. Stable C35 citrange tetraploids are becoming popular in high-density orchards.

The latest polyploidization trend involves polyploid characterization in their protocol regarding polyploid ultrastructure, bioactive compounds, photosynthetic capabilities, and metabolomics studies. Despite progress, we still lack a thorough understanding of polyploidization. Conflicting results have been reported for different polyploid species, and a single hypothesis cannot be proposed to explain plant polyploid evolution. Nevertheless, advances in sequencing technology, improved experimental analysis, multi-omics data quality, and more efficient analytical methods are likely to enhance our understanding of polyploidization in the near future significantly. In abiotic and biotic stress management cases, revealing the underlying mechanism is the most important research prospect of polyploidy. Polyploidy-based breeding combines the advantages of heterosis and apomixes, which can be a viable option for crop improvement in the future. A molecular approach to understanding the effects of polyploid plants on insects is necessary concerning polyploid-insect interaction.

## Figures and Tables

**Table 1:** Commonly used methods for polyploidization in vitro.

Plants	Treatment	Most Successful Method	References
**Vegetables**			
*Allium*	Callus	Colchicine 2.5 mM, 1/2 days	[60]
*Citrullus lanatus*	Germinating seedlings	2,6-Dinitroaniline 65.5 µM, 24 h	[61]
*Manihot esculenta*	Axillary node cuttings	Colchicine 5 mM, 48 h	[62]
*Smallanthus songifolius*	Nodal segments	Oryzalin 25 µM, 8–48 h	[63]
**Ornamentals**			
*Buddleja*	Nodal sections	Oryzalin 25 µM, 3 days	[64]
*Dieffenbachia*	Shoot clumps	Colchicine 1.25 mM, 24 h	[65]
*Dracaena deremensis*	Callus	Oryzalin 144.5 µM, 48 h	[66]
*Hypericum*	Callus	Oryzalin 30 µM, 3–9 days	[67]
*Lagerstroemia indica*	Nodal buds	Colchicine 750 µM 24 h	[68]
*Rhododendron*	Micro-shoots	Oryzalin 150 µM, 24 h	[69]
*Rosa*	Shoots tips, nodal sections	Oryzalin 5 µM, 1 day	[70]
*Rosa rugosa*	2 or 10mm nodes	Oryzalin 2.5 µM, 48 h	[71]
*Syringa*	Nodal sections	Colchicine 0.05–0.25 mM, 1–2 Days	[72]
*Alocasia*	Shoot tips	Oryzalin 289 µM, 24 h	[73]
*Alstroemeria*	Plantlets	Colchicine 5–15 mM, 6–24 h	[74]
*Cattleya*	PLB	Colchicine 1.25 mM, 8 days	[75]
*Cyclamen*	Tuber segments	Colchicine 0.25 mM, 4 days	[76]
*Lilium longiflorum*	Scale	Surflan (0.1 mM oryzalin), 3 h	[77]
*Tulipa gesneriana*	Flower stem dices	Oryzalin 2.88–120 µM, 2–24 h	[78]
*Watsonia lepida*	Shoots	Oryzalin 120 µM, 24 h	[79]
*Zantedeschia*	Shoot cultures	Colchicine 1.25 mM, 1–4 days	[80]
**Aromatic, medicinal plants**			
*Astragalus membranaceus*	Apical buds	Colchicine 5 mM, 36 h	[81]
*Bixa orellana*	Cotyledonary nodes from seedlings	Oryzalin 15 µM, 15 days	[82]
*Colophospermum mopane*	Seeds	Colchicine 2.5 mM, 48 h	[83]
*Dioscorea zingiberensis*	Apical buds	Colchicine 3.75 mM, 24 h	[53]
*Humulus lupulus*	Apical buds	Colchicine 1.25 mM, 48 h	[84]
*Zingiber officinale*	Shoot tips	Colchicine 5mM, 8 days	[85]

**Table 2:** Effect of polyploidization on plant morphology and yield attributes.

Induced Polyploid	Effect (Increased/Decreased)	References
Plant height	Increase	[52,54,115]
Root length and number	Increase	[86,116]
Number of leaves/plants	Increase/decrease	[117]
Leaf area	Increase/decrease	[17,118]
Leaf size	Increase	[17,112]
Stomata number/leaf	Decrease	[17,119,120]
Stomata size	Increase	[17,115,119]
Flower size, number	Increase	[52,112,117,118,119,120,121,122,123,124,125]
Pollen size	Increase	[123]
Fruit size, number	Increase	[98,116,124]
Seed size	Increase	[98,116]
Seeds/fruit	Decrease	[17,39]

**Table 3:** Effect of polyploidy on abiotic stress management.

Stress	Inducing Method/Organism	Crop	Adaptation	Mechanism	References
Salinity	NaCl induced salinity in laboratory	Orange	Better adaptation	1. NPK, proline content was higher in tetraploid than diploid. 2. MDA and H_2_O_2_ content was lower in tetraploid than in diploid.	[167]
		Turnip	Better adaptation	1. 100% increase in seed germination in tetraploid in highest saline level 200 (m.mol L^−1^). 2. Shoot and roots length reduced in diploid under salt stress condition compared to tetraploid. 3. At highest level of salinity, 74.7% diploid, and 64.4% tetraploid seedlings were injured. 4. Chlorophyll content reduced by 11.9% and 40.3% in tetraploid and diploid, respectively. 5. K^+^ concentration was stable in tetraploid (16:10, 15:10 K^+^/Na^+^ but reduced in diploid (46:100, 48:100) in root and shoot, respectively.	[165]
		Rice	Better adaptation	1. Proline concentration was higher in tetraploid (23.3% higher than diploid). 2. MDA content was lower in tetraploid than in diploid.	[163]
		Lemon Seedling	Better adaptation	1. Malondialdehyde and hydrogen peroxide was greater in the leaves and roots of diploid seedlings. 2. Antioxidative enzymes (peroxidase, ascorbate peroxidase, glutathione reductase, and catalase) were higher in tetraploid.	[167]
		Rice	Better adaptation	1. Mortality rates of tetraploids were lower than diploid. 2. Proline content was increased in tetraploid.	[164]
	*Hoagland* solution in green house pot	Citrus	Better adaptation	Lower accumulations of chloride ions in leaves of the tetraploid plants as compared to diploid.	[166]
Drought	Laboratory condition induced by polyethylene glycol	Apple	Better adaptation	1. Relative water content (RWC) was higher in tetraploid than diploid (after 3 h of treatment 81.76% and 63.84%, respectively, and after 6h of treatment 69.89% and 48.16%, respectively, in tetraploid and diploid cultivar). 2. Lower level of MDA content in tetraploid indicated membrane integrity under drought stress. 3. Less expression of aquaporin genes in drought stress was shown in tetraploid.	[168]
	Controlled environment, drought condition by limited water	A *solanaceous* plant	Better adaptation	1. Tetraploid plants grew normally, and leaves remained turgid where diploid plants died in drought stress. 2. Higher chlorophyll content and lower H_2_O_2_ synthesis were shown in tetraploid than diploid (less oxidative damage).	[169]
	Limited Water supply	*Arabidopsis*	Better adaptation	1. Tetraploid stomatal pore is 20% bigger than diploid due to the bigger size of the guard cells. 2. Higher survival rates in tetraploid. 3. ABA induced stomatal closure happened in tetraploid leaves. 4. ROS increased in cellular levels and affect stomatal aperture. 5. Polyploidy induced gene, which helps in stress adaptation.	[170]
	Limited Water supply	Rice	Better adaptation	1. MDA content was lower in tetraploid rice. 2. Phosphoenolpyruvate carboxylase (PEPC) alleviates photosynthesis inhibition. 3. Tetraploid showed more PEPC activities in drought stress. 4. Higher superoxide dismutase (SOD), POD (peroxidase), CAT (Catalase) was shown, ROS scavenging was more, and cell membrane damage was less in tetraploid rice.	[171]
	Both controlled and field trial	Westerwolths rye grass	Better adaptation	1. 30–40% more phenolic content and higher antiradical activities, better stress adaptation found in tetraploid. 2. More biomass in tetraploid.	[172]
	Laboratory	Honeysuckle plant	Better adaptation	1. No photosynthesis in diploid, 80% reduction in tetraploid. 2. Higher MDA in diploid.	[173]
Temperature	Heat Stress (42 °C)	*Dioscorea zingiberensis*	Better adaptation	1. Relative electrolyte leakage (%) and MDA content was lower in tetraploid than diploid in heat stress condition. 2. ROS production rate was higher in diploid and antioxidant enzymes such SOD, CAT, and APX were higher in tetraploid. 3. Glutathione-ascorbate and AsA declined slowly in tetraploid were drastically in diploid.	[123]
	Drought and Heat Stress (52 °C), field condition	Keystone grass	Better adaptation	1. 20% heavier seeds in tetraploid under stress condition. 2. Genome duplication and reproductive flexibility jointly contributes to stress alleviation. 3. Homeostatic maintenance of reproductive output under increasing abiotic stress. 4. Fixed differences in seed size and morphology that increase propagule fitness and mobility.	[174]
	Laboratory condition	*Dendranthema nankingense*	Lower heat stress adaptability	1. Higher cold stress adaptability in tetraploid but lower heat stress adaptability. 2. Tetraploid did not show much morphological change with diploid.	[175]
	Laboratory condition (39 °C day/30 °C night	*Dioscorea zingiberensis*	Better adaptation	1. Activation transcriptomic response in tetraploid (19 bands silenced and 47 bands activated) where in diploid 32 silenced and 28 activated. 2. Activation transcriptomic responses may confer tolerance in heat stress in tetraploid.	[176]
	96 h long stress at 45 °C.	*Asparagus officinalis*	Better adaptation	1. During heat stress MDA decreased by 42% in tetraploid, SOD increased by 81%, POD increased by 119%, and PRO content increased by 63% compared to diploid.	[177]

## Data Availability

Not applicable.

## References

[1] Adams K.L., Wendel J.F. (2004). Exploring the genomic mysteries of polyploidy in cotton. Biol. J. Linn. Soc..

[2] Lee J., Grant D., Vallejos C.E., Shoemaker R. (2001). Genome organization in dicots. II. *Arabidopsis* as a ‘bridging species’ to resolve genome evolution events among legumes. Theor. Appl. Genet..

[3] Udall J.A., Wendel J.F. (2006). Polyploidy and crop improvement. Crop Sci..

[4] Henry I.M., Dilkes B.P., Miller E.S., Burkart-Waco D., Comai L. (2010). Phenotypic consequences of aneuploidy in *Arabidopsis thaliana*. Genetics.

[5] Pavlíková Z., Paštová L., Münzbergová Z. (2017). Synthetic polyploids in *Vicia cracca*: Methodology, effects on plant performance and aneuploidy. Plant Syst. Evol..

[6] Shoemaker R., Polzin K., Labate J., Specht J., Brummer E., Olson T., Young N., Concibido V., Wilcox J., Tamulonis J.P. (1996). Genome duplication in soybean (*Glycine subgenus* soja). Genetics.

[7] Chai J., Su Y., Huang F., Liu S., Tao M., Murphy R.W. (2015). The gap in research on polyploidization between plants and vertebrates: Model systems and strategic challenges. Sci. Bull..

[8] Islam M.M., Yesmin R., Jung M.J., Kim H.Y., Kim C.K., Lim K.B. (2020). Investigation of the morphological and cytogenetic variations of an intraspecific Asiatic lily hybrid using 5S and 18S rDNA probes. Hortic. Environ. Biotechnol..

[9] Nei M., Nozawa M. (2011). Roles of mutation and selection in speciation: From Hugo de Vries to the modern genomic era. Genome Biol. Evol..

[10] Guo M., Davis D., Birchler J.A. (1996). Dosage effects on gene expression in a maize ploidy series. Genetics.

[11] Paterson A.H. (2005). Polyploidy, evolutionary opportunity, and crop adaptation. Genetica.

[12] Stuessy T., Weiss-Schneeweiss H. (2019). What drives polyploidization in plants?. New Phytol..

[13] Wittkopp P.J., Haerum B.K., Clark A.G. (2004). Evolutionary changes in cis and trans gene regulation. Nature.

[14] Clark R.M., Wagler T.N., Quijada P., Doebley J. (2006). A distant upstream enhancer at the maize domestication gene tb1 has pleiotropic effects on plant and inflorescent architecture. Nat. Genet..

[15] Endrizzi J., Turcotte E., Kohel R. (1985). Genetics, cytology, and evolution of *Gossypium*. Adv. Genet..

[16] Wendel J.F., Cronn R.C. (2003). Polyploidy and the evolutionary history of cotton. Adv. Agron..

[17] Bae S.J., Islam M.M., Kim H.Y., Lim K.B. (2020). Induction of Tetraploidy in Watermelon with Oryzalin Treatments. Hortic. Sci. Technol..

[18] Sattler M.C., Carvalho C.R., Clarindo W.R. (2016). The polyploidy and its key role in plant breeding. Planta.

[19] Gonzalez R.B., Miller C., Ramanna M., van Tuyl J. (2006). Nitrous oxide N_2_O incudes 2n gametes in sterile F_1_ hybrids of Oriental × Asiatic lilies (*Lilium*) and leads to intergenomic recombination. Euphytica.

[20] Jo Y.K., Mazharul I.M., Kim C.K., Kim H.Y., Lim K.B. (2019). Morphological Characteristics and FISH Analysis of *Hibiscus* F_1_ Hybrids and Parental Lines. Hortic. Sci. Technol..

[21] Lim K.B., Barba-Gonzalez R., Zhou S., Ramanna M., Van Tuyl J.M. (2008). Interspecific hybridization in lily (*Lilium*): Taxonomic and commercial aspects of using species hybrids in breeding. Floric. Ornam. Plant Biotechnol..

[22] Mazharul I., Reshma Y., Jung J., Mohammad D., Lim K. (2019). Cytogenetic assessment of *Lilium longiflorum* × *L. hansonii* revealed by genomic in situ hybridization (GISH). Acta Hortic..

[23] Van Tuyl J.M., Arens P. (2010). *Lilium*: Breeding history of the modern cultivar assortment. Acta Hortic..

[24] Ahmad P., Sharma S. (2008). Salt stress and phyto-biochemical responses of plants. Plant Soil Environ..

[25] Castillo E., To Phuc T., Ismail A.M., Inubushi K. (2007). Response to salinity in rice: Comparative effects of osmotic and ionic stresses. Plant Prod. Sci..

[26] Flowers T.J., Colmer T.D. (2008). Salinity tolerance in halophytes. New Phytol..

[27] Isayenkov S.V., Maathuis F.J. (2019). Plant salinity stress: Many unanswered questions remain. Front. Plant Sci..

[28] Coskun D., Britto D.T., Huynh W.Q., Kronzucker H.J. (2016). The role of silicon in higher plants under salinity and drought stress. Front. Plant Sci..

[29] Farooq M., Wahid A., Kobayashi N., Fujita D., Basra S. (2009). Plant drought stress: Effects, mechanisms and management. Agron. Sustain. Dev..

[30] Raja S., Ravikrishna R., Kommalapati R., Valsaraj K. (2005). Monitoring of fogwater chemistry in the gulf coast urban industrial corridor: *Baton Rouge* (*Louisiana*). Environ. Monit. Assess..

[31] Bita C., Gerats T. (2013). Plant tolerance to high temperature in a changing environment: Scientific fundamentals and production of heat stress-tolerant crops. Front. Plant Sci..

[32] Ghosh S.C., Asanuma K.I., Kusutani A., Toyota T.M. (2000). Leaf gas exchange properties of potato under different temperature and soil moisture at different growth stages. Environ. Control. Biol..

[33] Hatfield J.L., Prueger J.H. (2015). Temperature extremes: Effect on plant growth and development. Weather Clim. Extremes.

[34] Vollenweider P., Günthardt-Goergv M.S. (2005). Diagnosis of abiotic and biotic stress factors using the visible symptoms in foliage. Environ. Pollut..

[35] DeLucia E.H., Nabity P.D., Zavala J.A., Berenbaum M.R. (2012). Climate change: Resetting plant-insect interactions. Plant Physiol..

[36] Rasmann S., Pellissier L., Defossez E., Jactel H., Kunstler G. (2014). Climate-driven change in plant–insect interactions along elevation gradients. Funct. Ecol..

[37] Tobin P.C., Nagarkatt S., Loeb G., Saunders M.C. (2008). Historical and projected interactions between climate change and insect voltinism in a multivoltine species. Glob. Chang. Biol..

[38] Van de Peer Y., Maere S., Meyer A. (2009). The evolutionary significance of ancient genome duplications. Nat. Rev. Genet..

[39] Crow J.F. (1994). Hitoshi Kihara, Japan’s pioneer geneticist. Genetics.

[40] Touchell D.H., Palmer I.E., Ranney T.G. (2020). In vitro Ploidy Manipulation for Crop Improvement. Front. Plant Sci..

[41] Forrester N.J., Rebolleda-Gómez M., Sachs J.L., Ashman T.L. (2020). Polyploid plants obtain greater fitness benefits from a nutrient acquisition mutualism. New Phytol..

[42] Katepa-Mupondwa F.M., Christie B.R., Michaels T.E. (2002). An improved breeding strategy for autotetraploid alfalfa (*Medicago sativa* L.). Euphytica.

[43] Barton N.H. (2008). The role of hybridization in evolution. Mol. Ecol..

[44] Acquaah G. (2007). Principles of Plant Genetics and Breeding.

[45] Chen Z.J. (2010). Molecular mechanisms of polyploidy and hybrid vigor. Trends Plant Sci..

[46] Huang G., Zhu Y.X. (2019). Plant polyploidy and evolution. J. Integr. Plant Biol..

[47] Jiao Y., Wickett N.J., Ayyampalayam S., Chanderbali A.S., Landherr L., Ralph P.E. (2011). Ancestral polyploidy in seed plants and angiosperms. Nature.

[48] Salman Minkov A., Sabath N., Mayrose I. (2016). Whole-genome duplication as a key factor in crop domestication. Nat. Plants.

[49] Van Tuyl J., Lim K.B., Ramanna M., Vainstain A. (2002). Interspecific hybridization and introgression. Breeding for Ornamentals: Classical and Molecular Approaches.

[50] Gaul H. (1958). Present aspects of induced mutations in plant breeding. Euphytica.

[51] Winterfeld G., Ley A., Hoffmann M.H., Paule J., Röser M.J. (2020). Dysploidy and polyploidy trigger strong variation of chromosome numbers in the prayer-plant family (Marantaceae). Plant Syst. Evol..

[52] Simioni C., Schifino Wittmann M.T., Dall’Agnol M. (2006). Sexual polyploidization in red clover. Sci. Agric..

[53] Comai L. (2005). The advantages and disadvantages of being polyploid. Nat. Rev. Genet..

[54] Zhang K., Wang X., Cheng F. (2019). Plant Polyploidy: Origin, Evolution, and Its Influence on Crop Domestication. Hortic. Plant J..

[55] Otto S.P., Whitton J. (2000). Polyploid incidence and evolution. Annu. Rev. Genet..

[56] Dhooghe E., Van Laere K., Eeckhaut T., Leus L., Van Huylenbroeck J. (2011). Mitotic chromosome doubling of plant tissues in vitro. Plant Cell Tissue Organ Cult..

[57] Ramsey J., Schemske D.W. (1998). Pathways, mechanisms, and rates of polyploid formation in flowering plants. Annu. Rev. Ecol. Syst..

[58] Blakeslee A.F., Avery A.G. (1937). Methods of inducing doubling of chromosomes in plants: By treatment with colchicine. J. Hered..

[59] Chauvin J.E., Souchet C., Dantec J., Ellissèche D. (2003). Chromosome doubling of 2x Solanum species by oryzalin: Method development and comparison with spontaneous chromosome doubling in vitro. Plant Cell Tissue Organ Cult..

[60] Song P., Kang W., Peffley E.B. (1997). Chromosome doubling of *Allium fistulosum* × A. cepa interspecific F_1_ hybrids through colchicine treatment of regenerating callus. Euphytica.

[61] Omran S., Guerra Sanz J., Cardenas J.G. Methodology of tetraploid induction and expression of microsatellite alleles in triploid watermelon. Proceedings of the IXth Eucarpia Meeting on Genetics and Breeding of Cucurbitaceae.

[62] Awoleye F., Van Duren M., Dolezel J., Novak F. (1994). Nuclear DNA content and in vitro induced somatic polyploidization cassava (*Manihot esculenta* Crantz) breeding. Euphytica.

[63] Dunn B.L., Lindstrom J.T. (2007). Oryzalin-induced chromosome doubling in Buddleja to facilitate interspecific hybridization. HortScience.

[64] Henny R.J., Holm J.R., Chen J., Scheiber M. (2009). In vitro induction of tetraploids in *Dieffenbachia* × ‘Star Bright M-1′ by colchicine. HortScience.

[65] Teng E., Leonhardt K. (2007). In vitro and in vivo polyploidization of *Dracaena* with oryzalin. Acta Hortic.

[66] Meyer E.M., Touchell D.H., Ranney T.G. (2009). In vitro shoot regeneration and polyploid induction from leaves of *Hypericum* species. HortScience.

[67] Reshma Y., Mazharul I.M., Kim H.Y., Kim C.K., Lim K.B. (2020). Role of Growth Regulators in the Somatic Organogenesis of *Haworthia Inflorescences* in Vitro. Hortic. Sci. Technol..

[68] Zhang F., Xue H., Lu X., Zhang B., Wang F., Ma Y., Zhang Z. (2015). Autotetraploidization enhances drought stress tolerance in two apple cultivars. Trees.

[69] Kermani M., Sarasan V., Roberts A., Yokoya K., Wentworth J., Sieber V. (2003). Oryzalin-induced chromosome doubling in Rosa and its effect on plant morphology and pollen viability. Theor. Appl. Genet..

[70] Allum J., Bringloe D., Roberts A. (2007). Chromosome doubling in a *Rosa rugosa* Thunb. hybrid by exposure of in vitro nodes to oryzalin: The effects of node length, oryzalin concentration and exposure time. Plant Cell Rep..

[71] Rose J., Kubba J., Tobutt K. (2000). Induction of tetraploidy in *Buddleia globosa*. Plant Cell Tissue Organ Cult..

[72] Thao N.T.P., Ureshino K., Miyajima I., Ozaki Y., Okubo H. (2003). Induction of tetraploids in ornamental *Alocasia* through colchicine and oryzalin treatments. Plant Cell Tissue Organ Cult..

[73] Lu C., Bridgen M.P. (1997). Chromosome doubling and fertility study of *Alstroemeria aurea* × *A. caryophyllaea*. Euphytica.

[74] Xavier de Mello e Silva P.A.K., Callegari-Jacques S., Bodanese-Zanettini M.H. (2000). Induction and identification of polyploids in *Cattleya intermedia* Lindl. (*Orchidaceae*) by in vitro techniques. Cienc. Rural.

[75] Takamura T., Lim K.B., Van Tuyl J. (2001). Effect of a new compound on the mitotic polyploidization of *Lilium longiflorum* and Oriental hybrid *lilies*. Acta Hortic..

[76] Takamura T. (2007). Cyclamen. Flower Breed. Genet..

[77] Chauvin J., Label A., Kermarrec M. (2005). In vitro chromosome-doubling in tulip (*Tulipa gesneriana* L.). J. Hortic. Sci. Biotechnol.

[78] Ascough G.D., Van Staden J., Erwin J.E. (2008). Effectiveness of colchicine and oryzalin at inducing polyploidy in Watsonia lepida NE Brown. HortScience.

[79] Cohen D., Yao J.L. (1996). In vitro chromosome doubling of nine *Zantedeschia* cultivars. Plant Cell Tissue Organ Cult..

[80] Chen L.L., Gao S.L. (2007). In vitro tetraploid induction and generation of tetraploids from mixoploids in *Astragalus membranaceus*. Sci. Hortic.

[81] de Carvalho J.F.R.P., de Carvalho C.R.d.P., Otoni W.C. (2005). In vitro induction of polyploidy in annatto (*Bixa orellana*). Plant Cell Tissue Organ Cult..

[82] Rubuluza T., Nikolova R., Smith M., Hannweg K. (2007). In vitro induction of tetraploids in *Colophospermum mopane* by colchicine. S. Afr. J. Bot..

[83] Roy A., Leggett G., Koutoulis A. (2001). In vitro tetraploid induction and generation of tetraploids from mixoploids in hop (*Humulus lupulus* L.). Plant Cell Rep..

[84] Adaniya S., Shirai D. (2001). In vitro induction of tetraploid ginger (*Zingiber officinale* Roscoe) and its pollen fertility and germinability. Sci. Hortic..

[85] Alix K., Gérard P.R., Schwarzacher T., Heslop-Harrison J. (2017). Polyploidy and interspecific hybridization: Partners for adaptation, speciation and evolution in plants. Ann. Bot.

[86] Dewey D.R. (1980). Some applications and misapplications of induced polyploidy to plant breeding. Polyploidy.

[87] Van Tuyl J.M., Lim K.B. (2003). Interspecific hybridisation and polyploidisation as tools in ornamental plant breeding. Acta Hortic..

[88] Kamstra S.A., De Jeu M.J., Kuipers A.G., Jacobsen E. (1999). Homoeologous chromosome pairing in the distant hybrid *Alstroemeria aurea* × *A. inodora* and the genome composition of its backcross derivatives determined by fluorescence in situ hybridization with species-specific probes. Heredity.

[89] Lim K.B., Wennekes J., Jong J.H., Jacobsen E., Van Tuyl J.M. (2001). Karyotype analysis of *Lilium longiflorum* and *Lilium rubellum* by chromosome banding and fluorescence in situ hybridisation. Genome.

[90] Takahashi C., Leitch I., Ryan A., Bennett M., Brandham P. (1997). The use of genomic in situ hybridization (GISH) to show transmission of recombinant chromosomes by a partially fertile bigeneric hybrid, *Gasteria lutzii* × *Aloe aristata* (*Aloaceae*), to its progeny. Chromosoma.

[91] Veilleux R. (1985). Diploid and polyploid gametes in crop plants: Mechanisms of formation and utilization in plant breeding. Plant Breed Rev..

[92] Brownfield L., Köhler C. (2011). Unreduced gamete formation in plants: Mechanisms and prospects. J. Exp. Bot.

[93] Bretagnolle F.A., Thompson J.D. (1995). Gametes with the somatic chromosome number: Mechanisms of their formation and role in the evolution of autopolyploid plants. New Phytol..

[94] Lim K.B., Barba Gonzalez R., Zhou S., Ramanna M., Van Tuyl J.M. (2005). Meiotic polyploidization with homoeologous recombination induced by caffeine treatment in interspecific lily hybrids. Korean J. Genet..

[95] Mohammad D.D., Mazharul I.M., Ann T.C., Kim H.Y., Lim K.B. (2020). Phenotypic Characteristics and Karyotype Analysis of *Hibiscus sabdariffa* var. sabdariffa by Fluorescence in Situ Hybridization (FISH). Hortic. Sci. Technol..

[96] Duque R.E., Phan S., Hudson J.L., Till G., Ward P. (1985). Functional defects in phagocytic cells following thermal injury. Application of flow cytometric analysis. Am. J. Pathol.

[97] Sabharwal P., Doležel J. (1993). Interspecific hybridization in *Brassica*: Application of flow cytometry for analysis of ploidy and genome composition in hybrid plants. Biol. Plant.

[98] Suda J., Krahulcová A., Trávníek P., Krahulec F. (2006). Ploidy level versus DNA ploidy level: An appeal for consistent terminology. Taxon.

[99] He Y., Sun Y., Zheng R., Ai Y., Cao Z., Bao M. (2016). Induction of tetraploid male sterile *Tagetes erecta* by colchicine treatment and its application for interspecific hybridization. Hortic. Plant J..

[100] Younis A., Ramzan F., Hwang Y.J., Lim K.B. (2015). FISH and GISH: Molecular cytogenetic tools and their applications in ornamental plants. Plant Cell Rep..

[101] Andres R.J., Kuraparthy V. (2013). Development of an improved method of mitotic metaphase chromosome preparation compatible for fluorescence in situ hybridization in cotton. J. Cotton Sci..

[102] Lim K.B., De Jong H., Yang T.J., Park J.Y., Kwon S.J., Kim J.S. (2005). Characterization of rDNAs and tandem repeats in the heterochromatin of *Brassica rapa*. Mol. Cells.

[103] Gan Y., Liu F., Chen D., Wu Q., Qin Q., Wang C. (2013). Chromosomal Locations of 5S and 45S rDNA in *Gossypium* genus and its phylogenetic implications revealed by FISH. PLoS ONE.

[104] Wang Y., Wang X., Chen Q., Zhang L., Tang H., Luo Y. (2015). Phylogenetic insight into subgenera Idaeobatus and Malachobatus (*Rubus*, Rosaceae) inferring from ISH analysis. Mol. Cytogenet..

[105] Budylin M., Kan L.Y., Romanov V., Khrustaleva L. (2014). GISH study of advanced generation of the interspecific hybrids between *Allium cepa* L. and *Allium fistulosum* L. with relative resistance to downy mildew. Russ. J. Genet..

[106] Książczyk T., Taciak M., Zwierzykowski Z. (2010). Variability of ribosomal DNA sites in *Festuca pratensis*, *Lolium perenne*, and their intergeneric hybrids, revealed by FISH and GISH. J. Appl. Genet..

[107] Kwon M.J., Ramzan F., Ahn Y.J., Hwang Y.J., Kang Y.I., Kim C.K. (2017). Chromosomal analysis of *Lilium longiflorum* × Asiatic hybrids using GISH (genomic in situ hybridization). Hortic. Environ. Biotechnol..

[108] Ramzan F., Younis A., Lim K.B. (2017). Application of genomic in situ hybridization in horticultural science. Int. J. Genom..

[109] Allario T., Brumos J., Colmenero-flores J.M., Iglesias D.J., Pina J.A., Navarro L. (2013). Tetraploid Rangpur lime rootstock increases drought tolerance via enhanced constitutive root abscisic acid production. Plant Cell Environ..

[110] Hao G.Y., Lucero M.E., Sanderson S.C., Zacharias E.H., Holbrook N.M. (2013). Polyploidy enhances the occupation of heterogeneous environments through hydraulic related trade-offs in *Atriplex canescens* (*Chenopodiaceae*). New Phytol..

[111] Sun Q., Sun H., Li L., Bell R.L. (2009). In vitro colchicine-induced polyploid plantlet production and regeneration from leaf explants of the diploid pear (*Pyrus communis* L.) cultivar, ‘Fertility’. J. Hortic. Sci. Biotechnol..

[112] Levin D.A. (2004). The role of chromosomal change in plant evolution. Syst. Bot..

[113] Manzoor A., Ahmad T., Bashir M.A., Baig M.M.Q., Quresh A.A., Shah M.K.N. (2018). Induction and identification of colchicine induced polyploidy in *Gladiolus grandiflorus* ‘White Prosperity’. Folia Hortic..

[114] Vichiato M.R., Vichiato M., Pasqual M., Rodrigues F.A., Castro D.M. (2014). Morphological effects of induced polyploidy in *Dendrobium nobile* Lindl.(*Orchidaceae*). Crop Breed. Appl. Biotechnol..

[115] Khalid M.F., Hussain S., Anjum M.A., Ahmad S., Ali M.A., Ejaz S. (2020). Better salinity tolerance in tetraploid vs diploivolkamer lemon seedlings is associated with robust antioxidant and osmotic adjustment mechanisms. J. Plant Physiol..

[116] Ari E., Djapo H., Mutlu N., Gurbuz E., Karaguzel O. (2015). Creation of variation through gamma irradiation and polyploidization in *Vitex agnus-castus* L.. Sci. Hortic.

[117] Tan F.Q., Tu H., Liang W.J., Long J.M., Wu X.M., Zhang H.Y. (2015). Comparative metabolic and transcriptional analysis of a doubled diploid and its diploid citrus rootstock (*C. junos* cv. *Ziyang xiangcheng*) suggests its potential value for stress resistance improvement. BMC Plant Biol..

[118] Amah D., Van Biljon A., Maziya-Dixon B., Labuschagne M.T., Swennen R. (2019). Effects of in vitro polyploidization on agronomic characteristics and fruit carotenoid content; implications for banana genetic improvement. Front. Plant Sci..

[119] Prabhukumar K., Thomas V., Sabu M., Prasanth M., Mohanan K. (2015). Induced mutation in ornamental gingers (*Zingiberaceae*) using chemical mutagens viz. colchicine, acridine and ethyl methane sulphonate. J. Hortic. For. Biotechnol..

[120] Xue H., Zhang B., Tian J.R., Chen M.M., Zhang Y.Y., Zhang Z.H., Ma Y. (2017). Comparison of the morphology, growth and development of diploid and autotetraploid ‘Hanfu’apple trees. Sci. Hortic..

[121] Van Laere K., França S.C., Vansteenkiste H., Van Huylenbroeck J., Steppe K., Van Labeke M.C. (2011). Influence of ploidy level on morphology, growth and drought susceptibility in *Spathiphyllum wallisii*. Acta Physiol. Plant..

[122] Zhang Z., Kang X. (2010). Cytological characteristics of numerically unreduced pollen production in Populus tomentosa Carr. Euphytica.

[123] Liu S., Jiang Y., Guo X., Xu L., Lei P., Luo Q., Liu J., Li W., Tao L., Meng F. (2022). The lectin gene TRpL1 of tetraploid *Robinia pseudoacacia* L. response to salt stress. J. For. Res..

[124] Kobayashi N., Yamashita S., Ohta K., Hosoki T. (2008). Morphological characteristics and their inheritance in colchicine-induced *Salvia* polyploids. J. Jpn. Soc. Hortic. Sci..

[125] Ranney T.G. (2006). Polyploidy: From evolution to new plant development. Comb. Proc. Int. Plant Propagators’ Soc..

[126] Faria R.T.D., Takahashi L.S., Lone A.B. (2009). UEL 6: Nova cultivar de *Dendrobium*. Hortic. Bras..

[127] Schepper S.D., Leus L., Mertens M., Debergh P., Bockstaele E.V., Loose M.D. (2001). Somatic polyploidy and its consequences for flower coloration and flower morphology in azalea. Plant Cell Rep..

[128] Obute G.C., Ndukwu B., Chukwu O.F. (2007). Targeted mutagenesis in *Vigna unguiculata* (L.) Walp. and *Cucumeropsis mannii* (NAUD) in Nigeria. Afr. J. Biotechnol..

[129] Wu J.H., Ferguson A.R., Murray B.G., Jia Y., Datson P.M., Zhang J. (2012). Induced polyploidy dramatically increases the size and alters the shape of fruit in *Actinidia chinensis*. Ann. Bot..

[130] Samatadze T.E., Yurkevich O.Y., Khazieva F.M., Basalaeva I.V., Konyaeva E.A., Burova A.E., Zoshchuk S.A., Morozov A.I., Amosova A.V., Muravenko O.V. (2022). Agro-Morphological and Cytogenetic Characterization of Colchicine-Induced Tetraploid Plants of *Polemonium caeruleum* L. (Polemoniaceae). Plants.

[131] Khaing T., Perera A., Sumanasinghe V., Wijesundara D. (2007). Improvement of *Gymnostachyum* species by induced mutation. Trop. Agric. Res..

[132] Majdi M., Karimzadeh G., Malboobi M.A., Omidbaigi R., Mirzaghaderi G. (2010). Induction of tetraploidy to feverfew (*Tanacetum parthenium* Schulz-Bip.): Morphological, physiological, cytological, and phytochemical changes. Hort. Sci..

[133] Serrano-Fuentes M.K., Gómez-Merino F.C., Cruz-Izquierdo S., Spinoso-Castillo J.L., Bello-Bello J.J. (2022). Gamma Radiation (60Co) Induces Mutation during In Vitro Multiplication of Vanilla (Vanilla planifolia Jacks. ex Andrews). Horticulturae.

[134] Manzoor A., Ahmad T., Bashir M.A., Hafiz I.A., Silvestri C. (2019). Studies on colchicine induced chromosome doubling for enhancement of quality traits in ornamental plants. Plants.

[135] Niazian M., Nalousi A.M. (2020). Artificial polyploidy induction for improvement of ornamental and medicinal plants. Plant Cell Tissue Organ Cult..

[136] Rathod A., Patil S., Taksande P., Karad G., Kalamkar V., Jayade V. (2018). Effect of colchicine on morphological and biometrical traits in African marigold. J. Soils Crops.

[137] Jones K.D., Reed S.M., Rinehart T.A. (2007). Analysis of ploidy level and its effects on guard cell length, pollen diameter, and fertility in *Hydrangea macrophylla*. HortScience.

[138] Alexander L. (2020). Ploidy level influences pollen tube growth and seed viability in interploidy crosses of *Hydrangea macrophylla*. Front. Plant Sci..

[139] Cheniclet C., Rong W.Y., Causse M., Frangne N., Bolling L., Carde J.P. (2005). Cell expansion and endoreduplication show a large genetic variability in pericarp and contribute strongly to tomato fruit growth. Plant Physiol..

[140] Cohen H., Fait A., Tel Zur N. (2013). Morphological, cytological and metabolic consequences of autopolyploidization in *Hylocereus* (Cactaceae) species. BMC Plant Biol..

[141] Jokari S., Shekafandeh A., Jowkar A. (2022). In vitro tetraploidy induction in Mexican lime and sour orange and evaluation of their morphological and physiological characteristics. Plant Cell Tiss Org..

[142] Hassan J., Miyajima I., Ozaki Y., Mizunoe Y., Sakai K., Zaland W.J.P. (2020). Tetraploid induction by colchicine treatment and crossing with a diploid reveals less-seeded fruit production in Pointed Gourd (*Trichosanthes dioica* Roxb). Plants.

[143] Zlesak D.C., Thill C.A., Anderson N.O. (2005). Trifluralin-mediated polyploidization of Rosa chinensis minima (Sims) Voss seedlings. Euphytica.

[144] Cai X., Cao Z., Xu S., Deng Z. (2015). Induction, regeneration and characterization of tetraploids and variants in *Tapestry caladium*. Plant Cell Tissue Organ Cult..

[145] Luo Z., Iaffaldano B.J., Cornish K. (2018). Colchicine induced polyploidy has the potential to improve rubber yield in *Taraxacum koksaghyz*. Ind. Crops Prod..

[146] Pan-pan H., Wei Xu L., Hui Hui L., Xu X.Z. (2018). In vitro induction and identification of autotetraploid of *Bletilla striata* (Thunb.) Reichb. f. by colchicine treatment. Plant Cell Tissue Organ Cult..

[147] Ye Y., Tong J., Shi X., Yuan W., Li G. (2010). Morphological and cytological studies of diploid and colchicine-induced tetraploid lines of crape myrtle (*Lagerstroemia indica* L.). Sci. Hortic..

[148] Thatayaone M., Saji G., Meagle J., Kuruvila B. (2022). Biochemical and nutritional characteristics of some commercial banana (*Musa* spp.) cultivars of Kerala. Plant Sci. Today.

[149] Do Amaral C.M., Dos Santos-Serejo J.D.A., Silva S.D.O.E., Da Silva Ledo C.A., Amorim E.P. (2015). Agronomic characterization of autotetraploid banana plants derived from ‘*Pisang Lilin*’(AA) obtained through chromosome doubling. Euphytica.

[150] Jadrná P., Plavcová O., Kobza F. (2011). Morphological changes in colchicine--treated Pelargonium× hortorum LH Bailey greenhouse plants. Hortic. Sci..

[151] Manzoor S.A., Riaz A., Zafar T., Hassan M., Umar H.M., Hassan J., Alam W., Muhammad S., Mahmood M., Sohail H. (2016). Improving growth performance of jatropha curcas by inducing polyploidy through colchicine treatment. Am. J. Plant Sci..

[152] Li Z., Ruter J.M. (2017). Development and Evaluation of diploid and polyploid *Hibiscus moscheutos*. HortScience.

[153] Padoan D., Mossad A., Chiancone B., Germana M.A., Khan P.S. (2013). Ploidy levels in *Citrus clementine* affects leaf morphology, stomatal density and water content. Theor. Exp. Plant Physiol..

[154] Wong C., Murray B.G. (2012). Variable changes in genome size associated with different polyploid events in *Plantago* (*Plantaginaceae*). J. Hered..

[155] Lattier J., Chen H., Contreras R.N. (2019). Variation in genome size, ploidy, stomata, and rDNA signals in *Althea*. J. Am. Soc. Hortic. Sci..

[156] Yan J., Zhang J., Sun K., Chang D., Bai S., Shen Y. (2016). Ploidy level and DNA content of *Erianthus arundinaceus* as determined by flow cytometry and the association with biological characteristics. PLoS ONE.

[157] Wang J., Lei T., Meng F., Wei C., Li X., Guo H. (2019). Polyploidy index and its implications for the evolution of polyploids. Front. Genet..

[158] Nair N.V., Praneetha M. (2006). Cyto-morphological studies on three *Erianthus arundinaceus* (Retz.) Jeswiet accessions from the Andaman-Nicobar Islands, India. Cytologia.

[159] Manchanda G., Garg N. (2008). Salinity and its effects on the functional biology of legumes. Acta Physiol. Plant..

[160] Shahbaz M., Ashraf M. (2013). Improving salinity tolerance in cereals. Crit. Rev. Plant Sci..

[161] Hayat S., Hayat Q., Alyemeni M., Wani A., Pichtel J., Ahmad A. (2012). Role of proline under changing environments: A review. Plant Signal Behav..

[162] Tu Y., Jiang A., Gan L., Hossain M., Zhang J., Peng B. (2014). Genome duplication improves rice root resistance to salt stress. Rice.

[163] Jiang A., Gan L., Tu Y., Ma H., Zhang J., Song Z., He Y., Cai D., Xue X. (2013). The effect of genome duplication on seed germination and seedling growth of rice under salt stress. Aust. J. Crop Sci..

[164] Jiang J. (2019). Fluorescence in situ hybridization in plants: Recent developments and future applications. Chromosome Res..

[165] Meng H.B., Jiang S.S., Hua S.J., Lin X.Y., Li Y.L., Guo W.L. (2011). Comparison between a tetraploid turnip and its diploid progenitor (*Brassica rapa* L.): The adaptation to salinity stress. Agr. Sci. China.

[166] Saleh B., Allario T., Dambier D., Ollitrault P., Morillon R. (2008). Tetraploid citrus rootstocks are more tolerant to salt stress than diploid. C. R. Biol..

[167] Lloyd A., Bomblies K. (2016). Meiosis in autopolyploid and allopolyploid Arabidopsis. Curr. Opin. Plant Biol..

[168] Abdolinejad R., Shekafandeh A. (2022). Tetraploidy Confers Superior in vitro Water-Stress Tolerance to the Fig Tree (Ficus carica) by Reinforcing Hormonal, Physiological, and Biochemical Defensive Systems. Front. Plant Sci..

[169] Rao S., Tian Y., Xia X., Li Y., Chen J. (2020). Chromosome doubling mediates superior drought tolerance in *Lycium ruthenicum* via abscisic acid signaling. Hortic. Res..

[170] Del Pozo J.C., Ramirez-parra E. (2014). Deciphering the molecular bases for drought tolerance in *A rabidopsis* autotetraploids. Plant Cell Environ..

[171] Yang P.M., Huang Q.C., Qin G.Y., Zhao S.P., Zhou J.G. (2014). Different drought-stress responses in photosynthesis and reactive oxygen metabolism between autotetraploid and diploid rice. Photosynthetica.

[172] Akinroluyo O.K., Jasukune K., Kemesyte V., Statkeviciute G. (2020). Drought stress response of Westerwolths ryegrass (*Lolium multiflorum* ssp. *multiflorum*) cultivars differing in their ploidy level. Zemdirbyste.

[173] Li W.D., Biswas D.K., Xu H., Xu C.Q., Wang X.Z., Liu J.K., Jiang G.M. (2009). Photosynthetic responses to chromosome doubling in relation to leaf anatomy in *Lonicera japonica* subjected to water stress. Funct. Plant Biol..

[174] Godfree R.C., Marshall D.J., Young A.G., Miller C.H., Mathews S. (2017). Empirical evidence of fixed and homeostatic patterns of polyploid advantage in a keystone grass exposed to drought and heat stress. R. Soc. Open Sci..

[175] Liu S., Chen S., Chen Y., Guan Z., Yin D., Chen F. (2011). In vitro induced tetraploid of *Dendranthema nankingense* (Nakai) Tzvel. shows an improved level of abiotic stress tolerance. Sci. Hortic..

[176] Xu X., Lu J., Bradley F. (2010). Applications of polyploids in muscadine grape (*Vitis Rotundifolia* Michx.) breeding. Acta Hortic..

[177] Chen H., Lu Z., Wang J., Chen T., Gao J., Zheng J. (2020). Induction of new tetraploid genotypes and heat tolerance assessment in *Asparagus officinalis* L.. Sci. Hortic..

[178] Yin C., Li P., Li H., Xu L., Zhao J., Shan T. (2011). Enhancement of diosgenin production in Dioscorea zingiberensis seedling and cell cultures by beauvericin from the endophytic fungus *Fusarium* redolens Dzf2. J. Med. Plant Res..

[179] Arvanitis L., Wiklund C., Münzbergova Z., Dahlgren J.P., Ehrlén J. (2010). Novel antagonistic interactions associated with plant polyploidization influence trait selection and habitat preference. Ecol. Lett..

[180] Edger P.P., Heidel Fischer H.M., Bekaert M., Rota J., Glöckner G., Platts A.E. (2015). The butterfly plant arms-race escalated by gene and genome duplications. Proc. Natl. Acad. Sci. USA.

[181] Thompson J.N., Cunningham B.M., Segraves K.A., Althoff D.M., Wagner D. (1997). Plant polyploidy and insect/plant interactions. Am. Nat..

[182] Kao R.H. (2008). Implications of polyploidy in the host plant of a dipteran seed parasite. West. N. Am. Nat..

[183] Nuismer S.L., Thompson J.N. (2001). Plant polyploidy and non-uniform effects on insect herbivores. Proc. Royal Soc. B.

[184] Gross K., Schiestl F.P. (2015). Are tetraploids more successful? Floral signals, reproductive success and floral isolation in mixed-ploidy populations of a terrestrial orchid. Ann. Bot..

[185] Segraves K.A., Anneberg T.J. (2016). Species interactions and plant polyploidy. Am. J. Bot..

[186] Liu W., Yan Z., Zhao S., Gu Q., Li L. (2009). Study on Resistance to *Fusarium* Wilt in Different Polyploidy of Watermelons. J. Changjiang Veg..

[187] Gonda I., Milavski R., Adler C., Abu-Abied M., Tal O., Faigenboim A., Chaimovitsh D., Dudai N. (2022). Genome-based high-resolution mapping of fusarium wilt resistance in sweet basil. Plant Sci..

[188] Chen M., Wang F., Zhang Z., Fu J., Ma Y. (2017). Characterization of fungi resistance in two autotetraploid apple cultivars. Sci. Hortic.

[189] Gottula J., Lewis R., Saito S., Fuchs M. (2014). Allopolyploidy and the evolution of plant virus resistance. BMC Evol. Biol..

[190] Yuan J., Wang F., Liu T., Chen W. (2007). Expression and suppression of leaf rust resistance genes in amphidiploids from crosses of diploids and tetraploids. Acta Phytopathol. Sin..

[191] Zhu Z., Zhou R., Dong Y.C., Jia J. (2003). Analysis of powdery mildew resistance genes in some tetraploid wheat-*Aegilops amphidiploids* and their parents. Plant Genet. Resour..

[192] Coate J.E., Doyle J.J. (2010). Quantifying whole transcriptome size, a prerequisite for understanding transcriptome evolution across species: An example from a plant allopolyploid. Genome Biol. Evol..

[193] De Godoy L.M., Olsen J.V., Cox J., Nielsen M.L., Hubner N.C., Fröhlich F. (2008). Comprehensive mass-spectrometry-based proteome quantification of haploid versus diploid yeast. Nature.

[194] Te Beest M., Le Roux J.J., Richardson D.M., Brysting A.K., Suda J., Kubešová M. (2012). The more the better? The role of polyploidy in facilitating plant invasions. Ann. Bot.

[195] Mitchell S.E., Rogers E.S., Little T.J., Read A.F. (2005). Host-parasite and genotype-by-environment interactions: Temperature modifies potential for selection by a sterilizing pathogen. Evolution.

[196] Melaragno J.E., Mehrotra B., Coleman A.W. (1993). Relationship between endopolyploidy and cell size in epidermal tissue of *Arabidopsis*. Plant Cell.

[197] Olmo E. (1983). Nucleotype and cell size in vertebrates: A review. Basic Appl. Histochem.

[198] Pacey E.K., Maherali H., Husband B.C. Polyploidy increases storage but decreases structural stability in Arabidopsis thaliana. Current Biology.

[199] Petersen K.K., Hagberg P., Kristiansen K., Forkmann G. (2002). In vitro chromosome doubling of *Miscanthus sinensis*. Plant Breed.

[200] Pelé A., Rousseau-Gueutin M., Chèvre A.M. (2018). Speciation success of polyploid plants closely relates to the regulation of meiotic recombination. Front. Plant Sci..

[201] Bharadwa J., Dinesh N. (2015). Polyploidy in crop improvement and evolution. Plant Biology and Biotechnology.

[202] Mayer V.W., Aguilera A. (1990). High levels of chromosome instability in polyploids of *Saccharomyces cerevisiae*. Mutat. Res.-Fund. Mol. M..

[203] Klinner U., Böttcher F. (1992). Mitotically unstable polyploids in the yeast *Pichia guilliermondii*. J. Basic MicroBiol..

[204] Doyle G. (1986). Aneuploidy and inbreeding depression in random mating and self-fertilizing autotetraploid populations. Theor. Appl. Genet..

[205] Müntzing A. (2010). Cyto-genetic properties and practical value of tetraploid rye. Hereditas.

[206] Bomblies K., Madlung A. (2014). Polyploidy in the *Arabidopsis* genus. Chromosome Res..

[207] Papp I., Iglesias V., Moscone E., Michalowski S., Spiker S., Park Y.D. (1996). Structural instability of a transgene locus in tobacco is associated with aneuploidy. Plant J..

[208] Shi X., Chen C., Yang H., Hou J., Ji T., Cheng J., Veitia R.A., Birchler J.A. (2020). The Gene Balance Hypothesis: Epigenetics and Dosage Effects in Plants. Plant Epigenetics Epigenomics.

[209] Scheid O.M., Afsar K., Paszkowski J. (2003). Formation of stable epialleles and their paramutation-like interaction in tetraploid Arabidopsis thaliana. Nat. Genet..

[210] Scheid O.M., Jakovleva L., Afsar K., Maluszynska J., Paszkowski J. (1996). A change of ploidy can modify epigenetic silencing. Proc. Natl. Acad. Sci. USA.

